# Prediction of Under-Detection of Paediatric Tuberculosis in the Democratic Republic of Congo: Experience of Six Years in the South-Kivu Province

**DOI:** 10.1371/journal.pone.0169014

**Published:** 2017-01-06

**Authors:** Emmanuel André, Yvette Lufungulo Bahati, Eric Mulume Musafiri, Olivier Bahati Rusumba, Dimitri Van der Linden, Francis Zech

**Affiliations:** 1 Institut de Recherche Expérimentale et Clinique, Université Catholique de Louvain, Brussels, Belgium; 2 Cliniques Universitaires Saint-Luc, Brussels, Belgium; 3 Hôpital Provincial Général de Référence de Bukavu, Bukavu, Democratic Republic of Congo; 4 Coordination Provinciale Lèpre et Tuberculose, Bukavu, Democratic Republic of Congo; 5 Ambassadeurs de Lutte contre la Tuberculose, Bukavu, Democratic Republic of Congo; Foundation for Medical Research, INDIA

## Abstract

**Introduction:**

In the field of tuberculosis (TB), and particularly in regard to paediatric TB (PedTB), clinical skills of health professionals play an important role in determining quality of care. In an era where novel diagnostic technologies and efficient treatment regimens are being made available for the poorest, we must not divert our attention from the importance of clinical skills, as this deliverable remains the cornerstone of individualized patient care and ultimately the best assurance for optimal use of resources. The aim of our work was to study the epidemiology of PedTB and the determinants of PedTB under-detection in the South-Kivu Province of the Democratic Republic of Congo (DRC), a setting with nearly no technical resources allowing to support the clinical diagnosis of PedTB, i.e. chest X-rays, rapid molecular tests or culture laboratories.

**Methods:**

We collected TB notification data from 2010 to 2015 and analysed the space-time variations in notification for the different forms of TB among the 113 health facilities (HF) the South-Kivu Province, a region with a low HIV incidence. The different forms of TB notified were: smear positive pulmonary TB (SS+PTB), smear negative pulmonary TB (SS-PTB) and extra-pulmonary TB (EPTB). We further analysed the distribution of these different forms of the disease per age group and explored the possibility to predict the detection of PedTB.

**Results:**

Significant differences were observed between HF in regard to the proportion of paediatric TB and the proportion of SS-TB among adults. We found a strong correlation between the proportion of PedTB and three major factors: the proportion of TB cases with no bacteriological confirmation (SS-TB) among adults, the number of TB cases notified by the HF and the fact that the HF was supported or not by Médecins Sans Frontières (MSF). The proportion of SS-TB among adults was found to be a valid indicator for predicting the level of detection of PedTB at the same HF.

**Conclusion:**

Our observations strongly suggest that under-detection of PedTB is associated with insufficient clinical skills and technical resources at the HF level which similarly affects other forms of the disease, in particular SS-TB. We demonstrated that, in the specific context of South-Kivu, under-detection of PedTB can be predicted by a low SS-TB/SS+PTB ratio in the adult population. In the context of severely under-resourced settings, this ratio could be used to rapidly identify HF that should benefit in priority from deeper evaluation, and eventually targeted interventions.

## Introduction

The World Health Organisation (WHO) estimates that there were 9.6 million new patients infected with tuberculosis (TB) in 2014. Globally, an estimated 10% of TB patients are children less than 15 years, but two third of these are currently un-diagnosed or un-notified. The challenge of under-detection of TB is thus much more important among children than adults for which approximately one third of TB patients are currently missed [1]. Paediatric TB (PedTB) can rapidly evolve towards life-threatening manifestations such as miliary TB or meningitis [2]. Improving the capacity of health care providers to detect TB among children will not only save lives, but also have major repercussions in regard to the control of the epidemic.

The Democratic Republic of Congo (DRC) lies among the few high TB burden countries which did not meet any of the 2015 targets in regard to TB control: incidence is not falling and neither prevalence nor mortality was reduced by 50% in comparison with 1990 figures. In 2015, DRC notified 111,683 TB cases (146.8/100,000), roughly half of the estimated numbers of cases estimated for this country by WHO. In this low-income country, children aged less than 15 years represent 46% of the population compared to 26% globally [3]. Despite the over-representation of this age group, only 11.4% of TB patients were less than 15 years in 2015.

The aim of our work was to develop a method to rapidly identify health facilities (HF) which have a higher probability to be facing PedTB under-detection as a result of the inability of health-care providers to recognise this clinical entity. Such method would allow the design and implementation of targeted interventions, but also to evaluate their impact on PedTB detection.

## Materials and Methods

### Study setting

The DRC presents a very large network of HF constituting the public health system. TB-related services are well integrated within primary health care services, which offer free access to TB diagnostics—including rapid molecular tests in a minority of HF—and treatment [4].

Clinical guidelines developed by the National Tuberculosis Programme of DRC (17) include recommendations for the clinical diagnosis of PedTB. These recommendations are relatively scanty, as they take into account the current situation of the vast majority of HF which lack technical resources such as X-rays, molecular tests or tuberculin skin test (TST). Therefore, the diagnosis of PedTB is principally based on aspecific clinical symptoms and the notion of TB contact in the household. In practice, the use of rapid molecular diagnostic tests, which are being progressively scaled-up in the country since 2012, is currently limited to patients with presumptive drug resistant TB or HIV-TB coinfection. Due to this particular context, and considering that no drug resistance survey has been performed in DRC to date, the incidence of drug-resistant TB (and particularly among children) cannot be precisely estimated. Regular supervision activities are performed by the national and provincial teams, although some areas are inaccessible due to insecurity or the high costs associated with travelling in the most remote areas.

The South-Kivu province lies along the eastern border of DRC and presents a population of over 6,6 million inhabitants, mainly living in rural areas. Health delivery is provided to the population through over 500 HF, among which 78 (2010–2013) to 113 (2014–2015) provide TB services: Ziehl-Nielsen microscopy and provision of first-line anti-TB drugs. Since 2012, ten laboratories located in urban HF have been progressively equipped with GeneXpert MTB/RIF technology. The province does not have a laboratory able to perform mycobacterial cultures—the most sensitive diagnostic method—and diagnostic samples are usually not sent to the National Reference Centre in Kinshasa unless drug resistance is suspected.

The province is divided in 34 HZ, and each HZ has 1 to 6 HF. A HF is responsible for a median of 51,500 habitants (percentiles 10 and 90: 17,800 and 97,300). The number of TB cases notified by each HF is largely variable: 10% of these report less than 7 cases per year, while 10% report more than 140 cases, the median being 32. Among the 34 health zones (HZ), five are mainly urban (Bagira, Kadutu, Ibanda, Kamituga, and Uvira), while the remaining 29 HZ are located in rural areas. Some of the latter are relatively well served in terms of infrastructure and communications, while 11 are difficult to reach due to the absence of roads or on-going armed conflicts. Most of these isolated HZ are located in the centre and west of the province. As a result of the long-lasting humanitarian crisis in this region, the vast majority of the 34 HZ receive external support from non-governmental organisations (NGOs) for the delivery of primary health care services. This support is generally integrated within the existing public system and fully consistent with the national guidelines. Four HF located in the HZ Shabunda, Fizi and Kimbi-Lulenge (since 2012), are nevertheless considered as exceptions: these are supported by Médecins Sans Frontières (MSF) which applies a more “independent” policy in regard to the organisation of health services, including supplementation of the health structures with qualified medical staff, clinical guidelines and technical resources including laboratory equipment [5]. In regard to the diagnosis of PedTB, MSF recommends a documented growth assessment of the children suspected or exposed to TB. Furthermore, MSF supports sample transportation and provides technical resources such as X-rays, HIV testing, TST or investigations for EPTB. During the study period, there was no significant difference in the population covered by each HF between rural, isolated (enclaved), or MSF-associated HF (p = 0.14).

### Data collection and statistical analysis

We collected standard notification reports provided by the National TB Program. These reports include the notification of the different forms of TB, including smear positive pulmonary TB (SS+PTB), smear negative pulmonary TB (SS-PTB) and extra-pulmonary TB (EPTB). In this analysis, SS-PTB and EPTB among adults are considered jointly as smear-negative TB (SS-TB), while PedTB includes all forms of the disease among children under 15 years.

We analysed the changes in the incidence and proportion of different clinical TB presentations for each HF over a period of 6 years from 2010 to 2015. Each HF and each year was studied as a separate unit, with an internal correlation for the successive results of the same HF. The relations between the percentages of PedTB and diverse factors were examined using a generalised linear regression of a beta-binomial variable. Actually, the percentages of paediatric case followed a beta-binomial distribution, due to hyperdispersion of results between health areas. We used quasi-least squares, which is suitable for calculating internal correlations to adjust the regression, and chose the hypothesis of constant between-year correlation matrix. To calculate statistical significance, we used the “sandwich variance matrix” augmented by the bias correction proposed by Morel, Bokossa and Neerchal [6], which may be evaluated by the normal distribution. To study the relation between the percentages of PedTB cases with other factors, we first explored the curves of non-linear regressions and further selected the adequate expression to include these factors in linear univariate and multivariate regressions. We used the logarithm for the total number of TB cases reported for the populations surveyed by the HFs and the percentages of SS-TB adults. We report here only the results of linear regressions.

The data being anonymised, ethical approval was not required for this retrospective study.

## Results

### Notified incidence of TB

Between 2010 and 2015, the South-Kivu province reported 28,878 cases of TB. Among the 26,337 patients for which clinical outcome was available, 3.73% deceased during the year following diagnosis. This proportion was higher among SS-PTB (6.22%) and EPTB (4.89%) compared to SS+PTB (2.79%). During this period, the mean incidence of TB per 100,000 people per year was 84.5 new cases, among which 63.6% were adults with a pulmonary disease confirmed by microscopy. A transient increase in notification was observed due to the intensification of active case-finding strategies supported by two TB REACH grants [7].

The notified incidence was largely dependent on the notification of adults with SS+PTB, accounting for up to 100% of all TB notified in some HZ.

### Diagnosis of smear negative and extra-pulmonary TB among adults

Adults with SS-TB accounted for 21.8% of all cases notified and 28.5% of adult cases.

With regard to the yearly notified cases of SS-TB for each HZ during the 6 years, the percentage of SS-TB among adult cases was highly variable from one HF to the other. Moreover, we observed significant differences of the average percentages between rural HZ (15.7%), rural enclaved (19.4%), urban (37.5%), or MSF-supported HZ (34,7%).

### Diagnosis of paediatric TB

The proportion of children less than 15 years accounted for 14.6% of all TB cases notified in the South-Kivu province, compared to 11.4% at the national level (p<0.00001). In total, 4,219 PedTB cases were notified, among which 18.1% had a bacteriologically confirmed diagnosis and only two children were diagnosed with MDR-TB. In total, 56 MDR-TB cases were reported during the same period.

The proportion of PedTB cases notified varied significantly between HZ, with a ratio varying between 0% and 40%. This important variation cannot be explained only by demographic particularities, and rather suggests an important heterogeneity in the ability to diagnose PedTB between HF.

**Fig 1:**
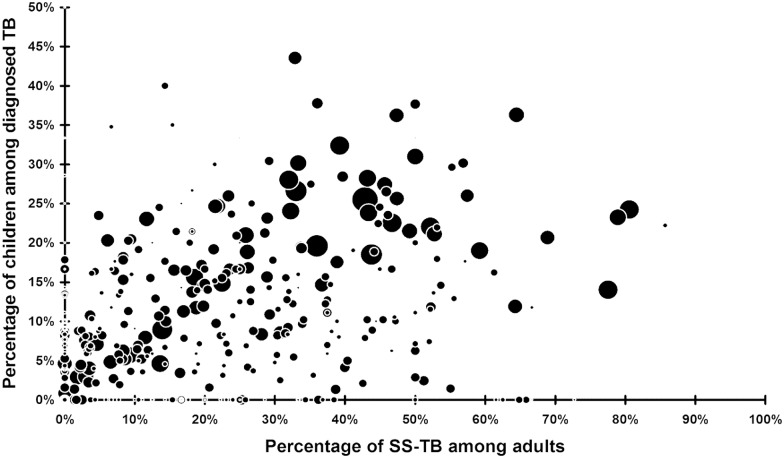
Correlation between the percentage of PedTB and the percentage of SS-TB among adults. Circles represent the number of TB cases notified for each Health Facility (HF) during one year (6 years are represented). A strong correlation (p<0.000001) was found between the proportion of SS-TB among adults and the proportion of PedTB.

Looking at the yearly notified cases of PedTB for each HF during the 6 years, we could not find any significant difference between urban, rural and isolated (enclaved) HZ (p = 0.074). The strongest association was found between the ratio of PedTB and the ratio of SS-PTB among adults (Fig 1) (p<0.000001). Furthermore, strong correlations were observed between PedTB detection and the total number of TB cases diagnosed (p = 0.000016) and with the presence of MSF in the HF (p<0.00001). The size of the population covered by each HF has no significant correlation with the ratio of PedTB.

### Development and validation of a model predicting under-detection of paediatric TB

**Fig 2:**
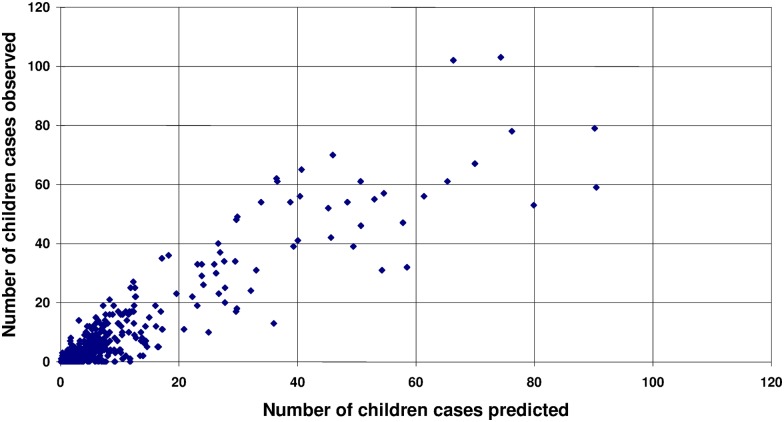
Application of the model predicting under-detection of PedTB at the HF level based on 3 informations: percentage of SS-TB among adults, external support received by MSF and the total number of TB cases notified by the HF during the year.

For the final multivariate linear regression, we used the results as a model to predict the number of PedTB cases for each HF and each year (Fig 2). This model is based on 3 variables easily available through the standard notification reports: the percentage of SS-TB among adults, the total number of TB cases notified by the HF during the year and an external support received by MSF. In the multivariate regression, these three factors remain independently significant: being in a HF with a low or great percentage of adults with SS-TB (p<0.000001), being or not in a MSF-supported HF (p<0.000001) and being in a HF with a low or great number of TB cases notified (p = 0.0011). The validity of this model was further confirmed by the analysis of the normalized Anscombe’s residues (data not shown).

## Discussion

### Particularities of the study setting and implication for TB surveillance

The South-Kivu province reports an incidence of 80.7/100,000 for all forms of TB, much lower than the 325/100,000 national incidence estimated by WHO. We found that the reported incidence is highly variable between HF, and particularly when looking at the different forms of the disease which are notified through quarterly reports. This observation suggests that there is a high inequality between HF in regard to the ability to diagnose complex clinical presentations of TB, including PedTB and SS-TB among adults. We could demonstrate with a strong level of evidence that the main factors which impact the detection of PedTB in South-Kivu, a setting with scarce technical resources, are linked with the clinical skills and the clinical experience of the health professionals. Indeed, the HF which receive from MSF an important input in terms of qualified health professionals tend to diagnose more PedTB and other complex forms of the disease. Furthermore, HF which diagnose many cases of TB (all forms of the disease), tend to be better skilled to recognise children affected by the disease. Of note, this critical importance of clinical skills may be exacerbated by the very poor infrastructure available in most HF: overall, only a very small proportion of TB suspects have access to X-ray, rapid molecular diagnostic or TST.

### Importance of clinical skills

The very strong association between under-detection of SS-TB among adults and under-detection of PedTB is therefore to be explained by the fact that recognizing these complex clinical entities require (particularly in the absence of performant radiological investigations and laboratory assays) higher clinical skills than the common smear positive pulmonary form of the disease. Interestingly, four rural HF supported by MSF had the highest proportion of TB cases diagnosed (mean 26.7%), and seemed to perform better than urban facilities in regard to PedTB detection. The explanation of these high figures are probably the result of highly qualified and adequately supported health staff and free access to X-rays or laboratory testing.

Insufficient clinical skills among health professionals has been reported as the main reason behind the frequent missed opportunities to diagnose and treat patients with TB that present to health facilities [8]. A qualitative study performed in Tanzania [9] reports that “inadequate awareness of the burden of childhood TB, limited knowledge of the wide spectrum of clinical presentation and lack of clinical decision support strategies is detrimental to the health staff's central responsibility of suspecting and referring children with TB especially in the early disease stages”. The study authors stressed the importance of implementing strategies focusing on awareness among health staff and clinical skills in order to improve diagnosis.

## Conclusion

There is a critical lack of medical doctors in rural areas of Africa. In 2013, the South-Kivu province counted 408 doctors (among which 14 had a specialization degree) and 2,862 nurses for a population of over 6,6 million. In such context, nurses, laboratory technicians or the so-called “Rapidly Trained Agents” form the front line of primary health-care delivery and are inevitably faced to medical conditions that they have not been trained to recognize. Furthermore, these professionals and the patients have very limited access to complementary tests such as X-Ray and accurate laboratory tests, which could eventually palliate part of these deficiencies.

The direct consequences of this lack of clinical skills are under-detection, increased mortality and continued transmission of the disease in the community [10]. As expected, SS-TB among adults are similarly affected by these insufficient clinical skills. In the South-Kivu province, these patients show an increased mortality during the first year after diagnosis.

In such context, performing a continuous assessment of all provider’s level of “knowledge, attitudes and practices (“KAP”) related to TB would be the best option, but is not something that can be implementable in many difficult contexts such as the rural health zones of the South-Kivu province. Our findings suggest that a first screening of the facilities which would beneficiate the most from a complete assessment can be done based on the—already collected—data. We propose to use the ratio between SS-TB and SS+TB notification among adults as an indicator for insufficient clinical skills and under-detection of TB among children.

This indicator should enable TB control programs and their partners to precisely and promptly identify those settings requiring additional support to tackle under-diagnosis of PedTB and other complex clinical presentations of the disease. The interventions should focus in parallel on the awareness of PedTB, active case finding strategies, strengthening of clinical skills oriented towards PedTB and finally the implementation of adequate technical resources such as X-rays, TST and rapid molecular laboratory assays. Improving clinical skills of health professionals should not be considered as the only priority, but the importance of these human skills should not be under-estimated as they remain the cornerstone of rational use of diagnostic and treatment resources. In DRC, availability of X-rays, TST, rapid diagnostic tests are still anecdotal when considering the thousands of health facilities located in rural areas. Furthermore, a recent study performed by the National Tuberculosis Programme revealed that only 47% of HF had access to anti-TB treatments with adapted paediatric drug formulation [11].

Continued efforts focusing on the quality of clinical skills are a keystone for tackling the dramatic burden of infectious diseases among children in low-resource settings [12–14]. We propose an innovative approach to rapidly identify those HF facing alarming indicators and which would beneficiate the most from additional human and technical resources.

## Supporting Information

S1 Fig Validity of the model predicting under-detection of PedTB. Graphical representation of normalized Anscombe-s residues.
(TIF)

S1 Dataset Dataset. (XLS)

## Abbreviations

DRC: Democratic Republic of Congo
EPTB: Extra pulmonary tuberculosis
HZ: Health Zone
HF: Health facility
INH: Isoniazid
MSF: Médecins Sans Frontières
MTB: *Mycobacterium tuberculosis* complex
NGO: Nongovernmental organization
PedTB: Paediatric tuberculosis
RIF: Rifampicin
SS+PTB: Smear-positive pulmonary tuberculosis
SS-PTB: Smear-negative pulmonary tuberculosis
SS-TB: Smear negative tuberculosis (SS-PTB and EPTB)
TB: Tuberculosis
WHO: World Health Organization
