# Supplementary figures and images for: Prediction of Under-Detection of Paediatric Tuberculosis in the Democratic Republic of Congo: Experience of Six Years in the South-Kivu Province
Source: PLoS One. 2017 Jan 6;12(1):e0169014. doi: 10.1371/journal.pone.0169014 (PMC5217857; doi:10.1371/journal.pone.0169014)

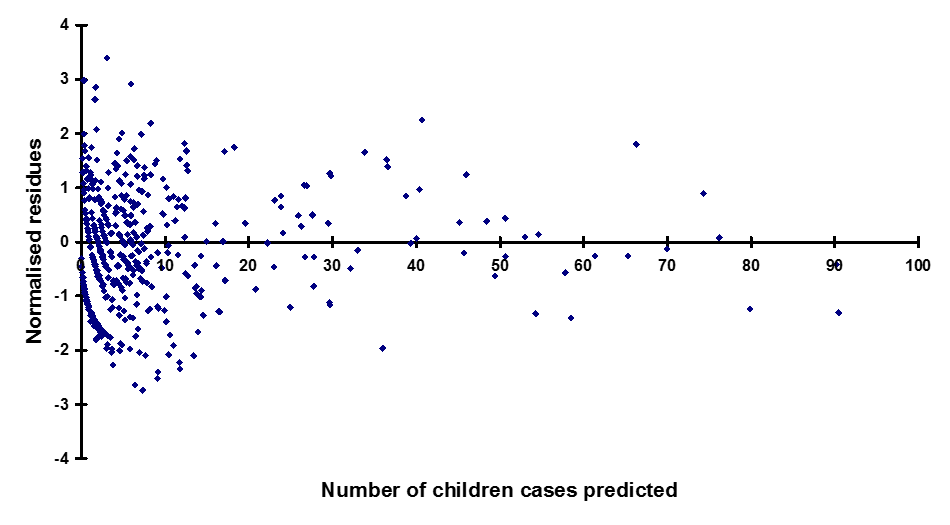

Supplement: S1 Fig — Graphical representation of normalized Anscombe-s residues. (TIF) [file pone.0169014.s001.tif]
